# Iterative Fragmentation of Cognitive Maps in a Visual Imagery Task

**DOI:** 10.1371/journal.pone.0068560

**Published:** 2013-07-17

**Authors:** Maryam Fourtassi, Abderrazak Hajjioui, Christian Urquizar, Yves Rossetti, Gilles Rode, Laure Pisella

**Affiliations:** 1 INSERM, U1028; CNRS, UMR5292; Lyon Neuroscience Research Center, Imp*Act* team, Lyon, France; 2 Université Lyon 1, Biologie Humaine, Lyon, France; 3 Hospices Civils de Lyon, Mouvement et Handicap, Hôpital Henry Gabrielle, St-Genis-Laval, France; 4 Mouvement et Handicap, Hôpital Neurologique, Lyon, France; 5 Faculté de Médecine et de pharmacie, Université Mohamed Premier, Oujda, Morocco; 6 Physical Medicine and Rehabilitation, CHU Hassan II Fez Faculty of Medicine and Pharmacy, University Mohammed Benabdellah, Fez, Morocco; Radboud University Nijmegen, The Netherlands

## Abstract

It remains unclear whether spontaneous eye movements during visual imagery reflect the mental generation of a visual image (i.e. the arrangement of the component parts of a mental representation). To address this specificity, we recorded eye movements in an imagery task and in a phonological fluency (non-imagery) task, both consisting in naming French towns from long-term memory. Only in the condition of visual imagery the spontaneous eye positions reflected the geographic position of the towns evoked by the subjects. This demonstrates that eye positions closely reflect the mapping of mental images. Advanced analysis of gaze positions using the bi-dimensional regression model confirmed the spatial correlation of gaze and towns’ locations in every single individual in the visual imagery task and in none of the individuals when no imagery accompanied memory retrieval. In addition, the evolution of the bi-dimensional regression’s coefficient of determination revealed, in each individual, a process of generating several iterative series of a limited number of towns mapped with the same spatial distortion, despite different individual order of towns’ evocation and different individual mappings. Such consistency across subjects revealed by gaze (the mind’s eye) gives empirical support to theories postulating that visual imagery, like visual sampling, is an iterative fragmented processing.

## Introduction

It has been observed that any mental activities are spontaneously accompanied by eye movements [1]. For example, mental arithmetic [2], response to questions [3] or memory recollection [1] is associated with eye movements. Since these eye movements occur even in the dark or with closed eyes [2], [4], they are not related to visual processing of the environment in which the cognitive task is performed but rather to the cognitive task itself, with the frequency of eye movements being correlated with the difficulty of the cognitive task [1]. It was hypothesized that the direction of eye movements was opposite to the cortical hemisphere engaged in the cognitive task (leftward in response to visuo-spatial questions and rightward in response to linguistic questions) but this has been discarded [5], [6].

The eye movements occurring during mental visual imagery have become a privileged research area because of the hypothesized analogy with the saccades and fixations sampling visual information during visual perception [7]. In addition, oculometric technics made it possible to experimentally test this analogy. Studies comparing eye movements or fixations between a perceptual encoding phase, in which subjects had to learn a new material, and a visual imagery phase, in which subjects had to recall details of this material, have confirmed this analogy [8]–[15]. Indeed, the eye movements evoked during visual imagery were not arbitrary. Instead, they have been shown to be similar to those observed during the encoding, or to reflect the content of the imagery, i.e. the spatial relationship between the different components of the material provided to the subject. These studies have led to the idea that eye movements may provide insights into the processes of visual imagery.

However, as long as visual imagery was preceded by an encoding phase during which subjects made eye movements, the eye movements measured during visual imagery might reflect the processes of memory retrieval [15]–[17] rather than visual imagery processes per se. Indeed, the recall from memory might activate the whole memory trace, including the eye movements to the corresponding location where a stimulus was encoded.

Several studies investigated the spontaneous eye movements accompanying visual imagery without a previous experimental encoding phase either during verbal description of a scene [10], [11] or during recall of geographical locations of French towns from long-term memory [18]. However, in the former, visual imagery was explicitly guided by spatial verbal indexes (e.g. “the tree to the left of the house”) and in the latter the visual imagery task consisted in stating whether a town given verbally by the experimenter was “left” or “right” of Paris. In both cases eye movements might reflect a reaction to the explicit spatial indexes of the task rather than the visual imagery processes themselves. Accordingly, such directional eye movements were observed when the subjects had no instructions to imagine anything but simply to listen to the verbal description [11].

Here, to address eye movements accompanying visual imagery without any preceding experimental encoding phase and without any explicit spatial indexes, we tested visual imagery from long-term memory in the following way. In our imagery condition, subjects had to imagine the map of France [18] and to name all the towns they visualize on this mental map [19], [20]. This task prevents from the direct recall of a common provided material and from the contamination of visual imagery from the frame and context, including external landmarks [21] of a preceding encoding phase. Instead, for natives of one’s country, towns’ names information has been encoded differently by each individual over the span of his/her life from various sources and scales (a variety of regional or national maps). Moreover, stored in long-term memory, this information belongs to semantic knowledge and is therefore not associated any more with a specific scanpath: town’s name can be evoked in different order, scales and strategies of retrieval (region by region with their administrative capital, or from the biggest town to the smallest…etc…).

However, since visual imagery cannot be fully dissociated from memory retrieval, we contrasted our imagery task with a control task of memory retrieval without imagery. Indeed, mental images are not built de novo; they necessarily reflect a combination of various sensorimotor experiences which might be stored in long-term memory. Roll et al. (1991) [22] postulated that the efferent commands to the eyes and proprioceptive information are stored along with the visual information. Mast & Kosslyn (2002) [23] guessed that such sensorimotor trace would presumably be preserved only in short-term memory but Martarelli & Mast (2012) demonstrated retention of this spatial information together with visual information after one week [17]. By contrasting trials with successful retrieval but unsucessful imagery and vice versa, neuroimaging techniques achieved to anatomically dissociate retrieval and imagery neural substrates [24]. However, visual imagery appears to be functionally tightly coupled with memory. Indeed, in addition to severe episodic memory deficits, patients with hippocampal damage show an impoverished ability to imagine fictitious events, even though these events never happened in their real lives [25]. It therefore appears impossible to convincingly study imagery without memory processes. Nevertheless, the imagery condition can be contrasted to a control condition of memory retrieval without imagery, because conversely, memory retrieval without visual imagery is possible. Contrary to most cognitive tasks in which sighted people can take advantage of the possibility to use visual imagery, in a phonological fluency task they do not perform better than early blind people [26]. Based on this experimental evidence that the phonological fluency task forces subjects to engage other strategies than visual imagery, we designed a task consisting in naming French towns starting with given letters. Moreover, this control (non-imagery) task was performed first, in order to avoid any contamination from the imagery experience.

**Figure 1:**
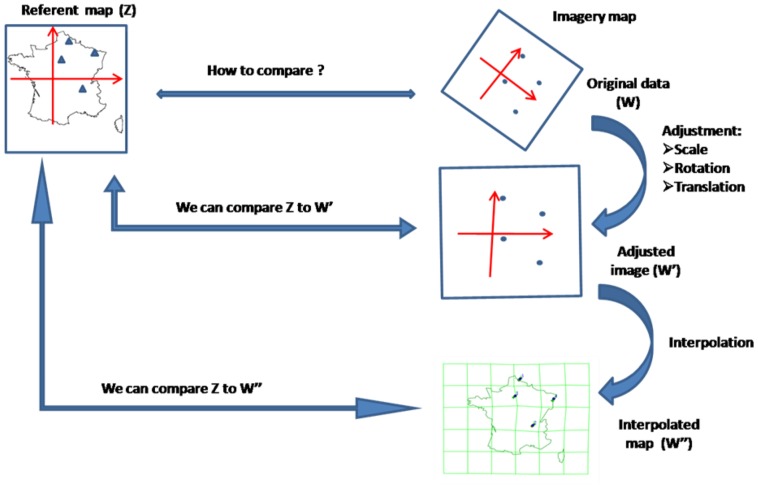
Schematic explanation of the bi-dimensional regression (BDR) according to the Euclidian model and its graphical representation using Darcy Software (inspired from [37]).

We also faced a challenge in terms of analysis because we did not provide any material to the subjects prior the experiment in order to neither constrain nor contaminate their mental activity. We aimed at comparing, in both imagery and non-imagery conditions, gaze location at the time of uttering each town and the real location of this town on the map of France according to the Global Positioning System (GPS). We first adapted spatial correspondence methods developed and validated in a previous study without encoding phase [10] and applied them to our data set. Secondly, we tested powerful statistical tools which have been developed specifically for comparing bi-dimensional data (like (X, Y) coordinates of eye positions). Bi-Dimensional Regression (BDR) is a statistic model, originally developed by Tobler (1965) [27] as a means of comparing the degree of resemblance between two planar representations of the same configuration, each defined in a different system of 2-dimensional coordinates, given a set of matching points in each representation. BDR models are also inference tools for identifying the transformation rules between two planes. BDR estimates the transformation function parameters based on a least-squares minimization and a goodness of fit measure defined as the bidimensional correlation coefficient (R) [28]. Widely acknowledged in geography, BDR has more recently revealed as a powerful tool in neurosciences for assessing the configural relations between cognitive and actual maps [29], i.e. for specifically studying the distortions in the mental representation of a given map. Choosing to use BDR, we postulated that the visual imagery map distortion as reflected by gaze positions may consist of a translation that brings the mean locations into coincidence, rotates the principle axis about this location, and/or produces a uniform change in scaling (Figure 1). BDR analysis would be resistant to such distortions and provide a powerful statistical tool to reveal correlations between gaze and town positions in imagery condition. With such tool, a lack of significant correlation in the non-imagery condition would strongly argue for eye movements being related to visual imagery specifically.

Finally, in his hypothesized analogy between visual imagery and visual perception, Hebb [7] intuitively suspected that eye movements in visual imagery would reflect a successive process of building mental images, since they are involved in sequentially sampling visual information during visual perception [30]. Although vision creates the impression that everything is perceived simultaneously, there is experimental evidence that the brain does not contain a ‘picture-like’ representation of the visual world that is stable and complete [31]. Vision instead implies multiple dynamic partial representations, given the restricted visual acuity and the limited number of elements that can be represented [32] and updated across saccades (review in [32], [33]). Like for active vision, one may expect that visual imagery would not consist in building a unique mental representation. Instead, visual imagery may involve the mapping of multiple successive images from memory. Accordingly, in a previous study where the same imagery task was used [34], our team observed that the same amount of towns were given with and without imagery but the localization of the successive towns, evoked in an imagery condition, was often characterized by the geographical proximity of neighboring towns in the series. Visual imagery may consist of building multiple successive partial representations of the map of France, each entailed with a specific spatial distortion (characterized by a new transformation function in the BDR model). Therefore, we aimed at studying the spatio-temporal dynamics of visual imagery through the evolution of the BDR coefficient of determination, as a function of the evoked towns’ sequence, for each subject.

## Materials and Methods

### Participants

Ten healthy subjects (5 men and 5 Women) volunteered to participate in the study. All of the subjects were French-natives and lived in France. All reported normal or corrected-to-normal vision.

Written informed consent was obtained from each subject before the experiment, which was conformed to the Code of Ethics of the World Medical Association (Declaration of Helsinki) and was approved by the local ethics committee of the Lyon Neuroscience Research Center (INSERM U1028 - CNRS UMR 5292).

### Apparatus

The eye tracker used was a SensoMotoric Instruments (SMI) iView pupil and corneal reflex imaging system with a sampling frequency of 200 Hz and spatial accuracy of 0.5°. It consisted of a scene camera and an eye camera mounted on a bicycle helmet. The outputs of the system were two temporally synchronised files: an MPEG video file and a data file providing gaze coordinates for each subject. A fixation was scored if the gaze remained stationary for at least 50 ms (ten consecutive measurement samples) [35] with a dispersion threshold of 1° on both X and Y coordinates.

### Procedure

Participants were comfortably seated in front of a white wall and were asked to keep their eyes open throughout the experiment. Head movements were restricted by an adjustable rest for the neck and nape. Participants were naïve about the real aim of the study and the recording of eye movements was not explicitly mentioned. They were told that the head-mounted camera measured their pupil dilatation which reflected their mental workload during effortful memory retrieval. A paper sheet with five dots defining a gaze calibration zone of 60 cm X 60 cm corresponding 30°x 30° was sticked on the wall (at 114 cm from the subject) and immediately removed after calibration. Then, the participants were asked to perform the control task followed by the imagery task.

#### The phonological fluency task (control task)

This task was meant to lead to memory retrieval without visual imagery. The participants received the following instruction: *“When you hear the starting signal, give as many French towns as you can whose names begin with the letter you will hear. If you can find no more French towns’ names beginning with the first given letter and want to change letter then say “change” and you will hear the next letter.”* The given letters were “A”, “P”, “B”, “M”, “L”, “C”, “S”, “R” in the same order for all the subjects, who often did not go through all the letters because the recording was stopped after two-minute duration for every subject. These letters were specifically chosen for this task because they were initials of a substantive number of large French towns.

#### The visual imagery task

In this task, participants were given the following instructions. *“Now, imagine a map of France. When you hear the starting signal, give the maximal number of French towns you can visualize on your imagined map.”* Also in this task, the recording was stopped after two-minute duration for every subject. If the subjects spontaneously stopped before the two minutes, they were asked to give more towns.

### Analysis

The sound file of the sequence of towns uttered by each subject was extracted from the mpeg video file and used to determine the precise time of each verbal response. To determine the eye-position corresponding to a town name, we searched for the fixation occurring in a temporal range of 2 seconds, before or after the town name was pronounced. When more than one town name was pronounced in the 4 seconds range, then the interval was shortened to avoid any overlap (the timing border was set in between the two successive town names). When there was more than one fixation in the set time interval, the fixation of longer duration was systematically considered.

#### Spatial correspondence analysis

**Figure 2:**
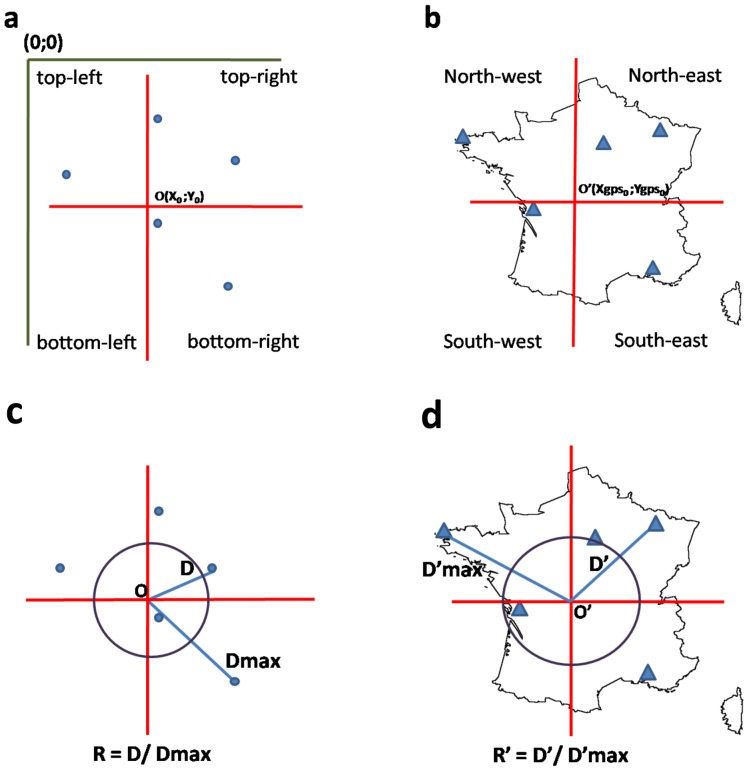
Spatial correspondence analysis. 1) Grouping in four quadrants according to the reference center for both systems of coordinates; the gaze coordinates (a) and the GPS coordinates (b). 2) Determining the degree of remoteness from the reference centre for each system of coordinates; gaze coordinates (c) and GPS coordinates (d). The circle represents a 50% degree of remoteness from the centre according to Dmax.

This analysis was largely inspired from a previous study by Johansson et al. (2006). Correspondence of the eye movements was analyzed for all the towns pronounced by each subject in both tasks using a method to assess the positions of the eye within the subject’s entire gaze pattern (scanpath). To this purpose, we defined for each scanpath, a reference central point O (X0, Y0) with X0 = (Xmax-Xmin)/2 and Y0 = (Ymax-Ymin)/2. Then, we normalized the gaze coordinates according to this new reference center and did the same operation for the GPS coordinates according to the centre of the map of France O’ (Xgps0, Ygps0). This new reference center identified 4 quadrants for each system of coordinates (See Figure 2 (a) and (b)).

The correspondence of each two pairs of coordinates (gaze coordinates and GPS coordinates) for a given town was determined in terms of direction only (low correspondence) and in terms of both direction and amplitude (high correspondence). To achieve low correspondence, the gaze location for a given city had to be localized in the same quadrant as the GPS location on the real map. High correspondence was achieved if the gaze location was not only in the same quadrant, but also within the same degree of remoteness from the centre of reference. As subjects might visualize the map using different scales, we defined for each town the relative remoteness (R) from the reference centre. R is the ration D/Dmax, with D being the distance between a given town’s coordinates and the reference centre coordinates, and Dmax the distance between this reference centre and the furthest town mentioned. Here we took as distance ratio cut-off R = D/Dmax = 0.5. Thus gaze location and GPS location were considered as having an equal distance from the reference centre if they were both inside the circle or both outside the circle (See Figure 2 (c) and (d)).

The number of fixations scored correct according to low and high correspondence was then compared with the possibility that the participant’s fixation would lie at the correct position by chance. For low correspondence, the probability for the eye to be located in the correct quadrant by chance was 1/4 (25%). For high correspondence, the probability for the eye to be located in both the correct quadrant (1/4) and the correct remoteness (1/2) from the center by chance was defined as 1/8 (12.5%). These percentages of chance were transformed into individual expected number of correct occurrence that would be made by chance based on the number of towns provided for each subject. These two paired samples (observed number versus number expected from chance) were then analyzed using Wilcoxon signed-ranks test [36] for low and high correspondence separately.

#### Bi-dimensional regression (BDR) analysis

To verify our findings at an individual level, we compared for each subject the gaze locations at the time of uttering each town and the real GPS locations of these towns using the BDR model [27]. The BDR is based on the same statistical principles as the one-dimensional regression with a major difference that it applies to bi-dimensional variables (X;Y) systems [29]. The Eucledian BDR model is characterized by the following equation where (A;B) are the dependent variables (the image) and (X;Y) the independent variables (the source).
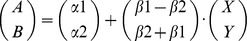



The intercept has two components α2 and α1 reflecting the vertical and horizontal translation factors, respectively. The slope has two components β1 and β2 which are used to compute the scale transformation magnitude ф = (β1+ β2)^1/2^ and the rotation angle θ = tan^−1^(β2/β1) by which the original coordinates were transformed to derive the least square fit [29].

Using BDR Matlab application developed by TJ Pingel (http://www.geog.ucsb.edu/), we investigated the correlations between the gaze locations at towns’ evocation (dependent variables, variant map) and the longitudes and latitudes of these towns, converted to planar (X;Y) coordinates (independent variables, referent map). Like uni-dimensional regression, the BDR provides for each subject a correlation coefficient (R) and a p-value according to the test F for regression.

#### Chronological evolution of the BDR coefficient of determination

The BDR coefficient of determination (R^2^) reflects the goodness of fit of the regression model. We studied for each subject, the evolution of the value of the R^2^ based on the number of cities mentioned in their chronological order, to determine whether the correlation either gained or lost strength and when more towns (data, potential errors) were added. The dynamics of R^2^ evolution of each subject were compared in order to determine whether a common pattern could be identified.

### Graphic Representations of the Mental Maps as Reflected by Gaze Positions

Graphical representations of bi-dimensional regressions were realized using Darcy®2.0 software [37]. This software extracts graphics after a two-step process: 1) the “adjustment” between the two systems of coordinates (gaze and GPS coordinates) according to the BDR parameters (translations, scale adaptation and rotation) is calculated on the observed data points, and 2) the “interpolation” extends the adjustment algorithms to the entire studied area (here the map of France) in order to obtain an illustration of the mapping distortion. The interpolation process involves superimposing a grid on the adjusted image in order to obtain values at any point of the map surface.

## Results and Discussion

### Descriptive Results

The number of town provided by each subject in the two tasks is displayed on Table 1. In most of the subjects, but not all, the number of towns given in the visual imagery condition was higher than in the phonological fluency task. This might be explained either by the order of the tasks (the visual imagery task performed at the end could have benefited from the previously retrieved towns’ names) or by a possible facilitation of memory retrieval by visual imagery processes [38].

**Table 1:** Total number of given town names in the two tasks.

Subject number	Imagery Task	Control task
1	25	11
2	66	30
3	10	15
4	27	19
5	40	20
6	36	14
7	38	37
8	35	20
9	44	15
10	31	15
Total Number	352	196

### Spatial Correspondence Analysis

In the imagery task, the correspondence between eye-fixations and real GPS locations of the uttered French towns was significantly different from chance levels in both low and high correspondence models (Table 2), whereas neither low nor high correspondence reached significance in the control task (Table 3). Although our adaptation of the spatial correspondence analysis was based on the idea that gaze would faithfully match a unique static mental representation of the true map of France, the correspondence was strong enough to reach the high level in the visual imagery task and not even the low level in the phonological task. As memory retrieval of French town names was present in the two tasks, this result demonstrated at the group level that the spatial correspondence between gaze and the towns’ positions was specifically related to visual imagery.

**Table 2:** Difference between observed and chance-expected correct eye positions, in the visual imagery task.

Eye position coding	% of correct eye positions	Statistical significance Wilcoxon signed rank statistic
Low correspondance	35	W = 52, z = −2.65, p = 0.008
High correspondance	18.5	W = 50, z = −2,54, p = 0.01

**Table 3:** Difference between observed and chance-expected correct eye positions, in the phonological fluency task.

Eye position coding	% correct eye positions	Statistical significance Wilcoxon signed rank statistic
Low correspondance	25	W = 2, z = −0.10, p = 0.20
High correspondance	9.5	W = 5, z = −1.27, p = 0.92

### BDR Analysis

To verify our findings at an individual level, we compared for each subject the mental map, as reflected by gaze locations at each town name’s uttering, and the real GPS map using the BDR model. BDR performed in each subject strongly confirmed the preceding group analysis. A significant correlation (all p<0.05) was found between the mental and the real map for every single subject in the imagery task and for none of the individuals in the control task (all p>0.05) (See Table 4). In other words, no subject reported the town names without positioning the eyes in relation to their geographic location when asked to visualize them on a mental map. Conversely, despite their incessant eye movements and the statistical power of the BDR, no subject presented a spatial correlation between their eye positions and the town geographical locations while reporting town names through phonological access. These clear-cut results not only validate the BDR as a method to study eye positions during visual imagery but also provide a specific link between gaze location and visual imagery at an individual level. Several authors have postulated a functional role of eye movements in visual imagery [7], [9], [18], [39], [40]. However, as also mentioned by these authors, it might not necessarily be the eye movements per se, but the processes that drive them, which are functionally associated with the construction of visual images. In other words, if the construction of visual images is reflected by overt ocular behavior in our task, this does not necessarily imply a functional role played by the saccadic execution per se. The functional role might rather be attributed to the processes of saccade planning which have been often assimilated to covert shifts of attention (for discussions about the functional coupling between saccade planning and covert attention but their possible neural dissociation see [41]–[45]). The possibility to perform simple imagery tasks when participants are instructed to withhold eye movements suggests that covert attention shifts may be sufficient [46], [40]. Participants may draw primarily on transformational processes or on attentional processes to scan a mental image [47].

**Table 4:** Statistical significance (F test) and BDR coefficient of correlation (R) in each subject, in the two experimental conditions, with gaze locations being the dependent variables and towns’ GPS positions being the independent variables.

Subjects	Phonological fluency task		Visual imagery task	
	p-value	R	p-value	R
1	0.823	0.14	0.0001	0.61
2	0.322	0.17	0.05	0.39
3	0.803	0.1	0.0001	0.87
4	0.562	0.17	0.0001	0.67
5	0.503	0.17	0.0001	0.55
6	0.185	0.36	0.0001	0.51
7	0.685	0.14	0.0001	0.48
8	0.461	0.2	0.0001	0.85
9	0.546	0.2	0.0001	0.68
10	0.450	0.22	0.01	0.36

Saccades might or might not accompany these processes. This would explain why the coefficient of determination (R^2^), which reflects the proportion of variability in a data set that is accounted for by the statistical Euclidian model, remained relatively low in most subjects even if the correlations were significant.

An additional explanation would be that a unique Euclidian transformation function was calculated for the entire duration of the visual imagery task. The analysis can be further improved if we consider that visual imagery may not consist in building a unique mental representation. Instead, it may consist of building multiple successive partial representations of the map of France, each entailed with a specific spatial distortion (characterized by a new transformation function in the BDR model).

### Fragmentation of Visual Imagery

**Figure 3:**
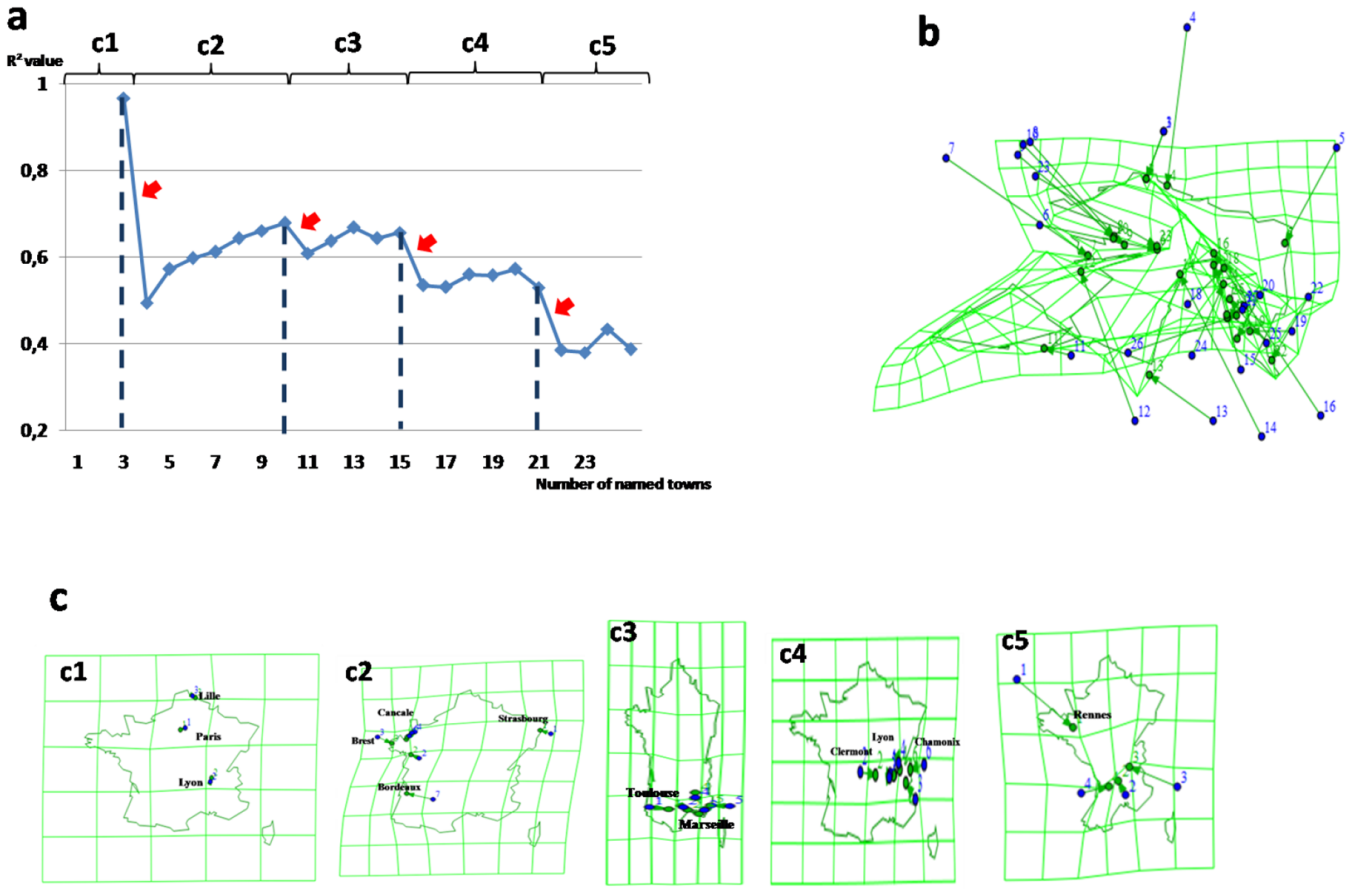
Graphic representation of the cognitive map of France as reflected by gaze positions for the subject n°1, after adjustment and interpolation, according to BDR and using Darcy software. (a) The coefficient of determination (R^2^) of BDR in the subject n°1, in the imagery condition, according to the number of towns evoked in chronological order. The curve shows 4 drastic drops pointed by arrows. (b) Representation of all the towns evoked by the subject n°1 during the 2 minutes duration of the imagery task, where the green points correspond to the adjusted gaze positions and the blue points represent the real GPS positions of the same towns. (c) Graphic representations of gaze positions, limited to small sequences of towns in their chronological order, in the same subject. The cut-off between these sequences was defined by the abrupt decreases in the R^2^ curve.

Congruent with the above prediction, we observed that R^2^ values decreased as a function of the evoked towns’ sequence, for each subject with a stereotyped pattern. This diminution was not progressive but rather characterized by plateaus and drastic drops (red arrows on Figure 3a for a typical subject; see other subjects on Figures S1, S2, S3, S4, S5, S6, S7, S8, S9). The drops occurred about every 6 successive towns on average, defining series of towns mapped with similar spatial distortion (plateau in R^2^ values) in-between. This finding reveals that imagery of the map of France was not generated at once as a global and unique mental image. Instead, imagery appears to be made up of a succession of partial mental images. The transition between two partial images may be revealed objectively by the drops in the R^2^ function across the order of towns evoked. Each drop would represent a change in one or more of the BDR adjustment parameters, i.e. a translation, a rotation or a scale variation at each new partial image generation rather than a decrease of spatial correlation. Each successive partial mapping can be objectified by illustrations provided by Darcy® 2.0 Software [37]. The variations of scales and orientations between successive maps are illustrated on Figure 2c for a typical subject (see other subjects on Figures S1–S9). The illustration of a unique visual representation produced for the entire sequence of towns is provided for a typical subject on Figure 3b. Even if the spatial correlation is significant for each subject, the graphic does not appear visually consistent with the real map of France. For comparison, Figure 3c (see other subjects on Figures S1–S9) presents the successive representations separated by the R^2^ drops, which clearly show an improved visual consistency with the real map of France. Since this improved consistency was found for each subject, it provides a strong argument for a common fragmentation procedure of visual imagery, in which eye movements allow one to visualize a montage, a composite created from multiple and various memories [23].

### Conclusion

To sum up, spatial correspondence between the sequence of gaze and town locations was revealed only when visual imagery accompanied memory retrieval. As reflected by the evolution of the BDR coefficient of determination with the number of towns reported, all subjects used a common formula, i.e. iteratively generated a series of partial mental images, each of them representing a limited number of towns. BDR graphical representations revealed that this common sequential procedure of visual imagery between individuals did not prevent each individual from exhibiting different spatial strategies of town evocation and different spatial distortions. Therefore, gaze recording and BDR analysis are powerful tools to both reveal the common dynamics and procedures of visual imagery and study specific individual imagery distortions, which could be interesting, especially following brain damage (e.g. representational neglect: [34]).

## Supporting Information

Figure S1 Graphic representation of the cognitive map of France as reflected by gaze positions, in the imagery task, for the subject n°2.
(TIF)

Figure S2 Graphic representation of the cognitive map of France as reflected by gaze positions, in the imagery task, for the subject n°3.
(TIF)

Figure S3 Graphic representation of the cognitive map of France as reflected by gaze positions, in the imagery task, for the subject n°4.
(TIF)

Figure S4 Graphic representation of the cognitive map of France as reflected by gaze positions, in the imagery task, for the subject n°5.
(TIF)

Figure S5 Graphic representation of the cognitive map of France as reflected by gaze positions, in the imagery task, for the subject n°6.
(TIF)

Figure S6 Graphic representation of the cognitive map of France as reflected by gaze positions, in the imagery task, for the subject n°7.
(TIF)

Figure S7 Graphic representation of the cognitive map of France as reflected by gaze positions, in the imagery task, for the subject n°8.
(TIF)

Figure S8 Graphic representation of the cognitive map of France as reflected by gaze positions, in the imagery task, for the subject n°9.
(TIF)

Figure S9 Graphic representation of the cognitive map of France as reflected by gaze positions, in the imagery task, for the subject n°10.
(TIF)
