# Supplementary figures and images for: Iterative Fragmentation of Cognitive Maps in a Visual Imagery Task
Source: PLoS One. 2013 Jul 17;8(7):e68560. doi: 10.1371/journal.pone.0068560 (PMC3714244; doi:10.1371/journal.pone.0068560)

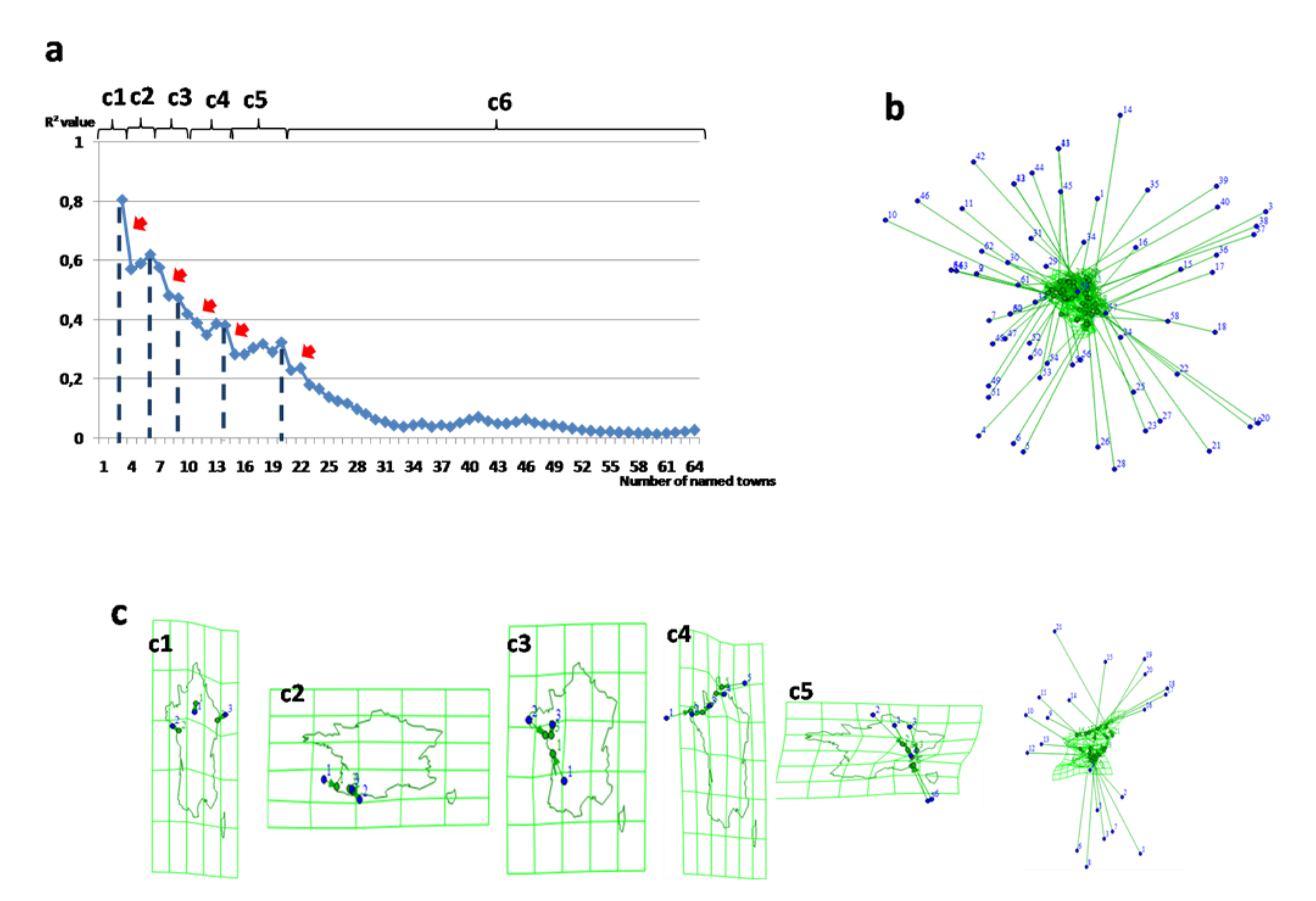

Supplement: Figure S1 — Graphic representation of the cognitive map of France as reflected by gaze positions, in the imagery task, for the subject n°2. (TIF) [file pone.0068560.s001.tif]

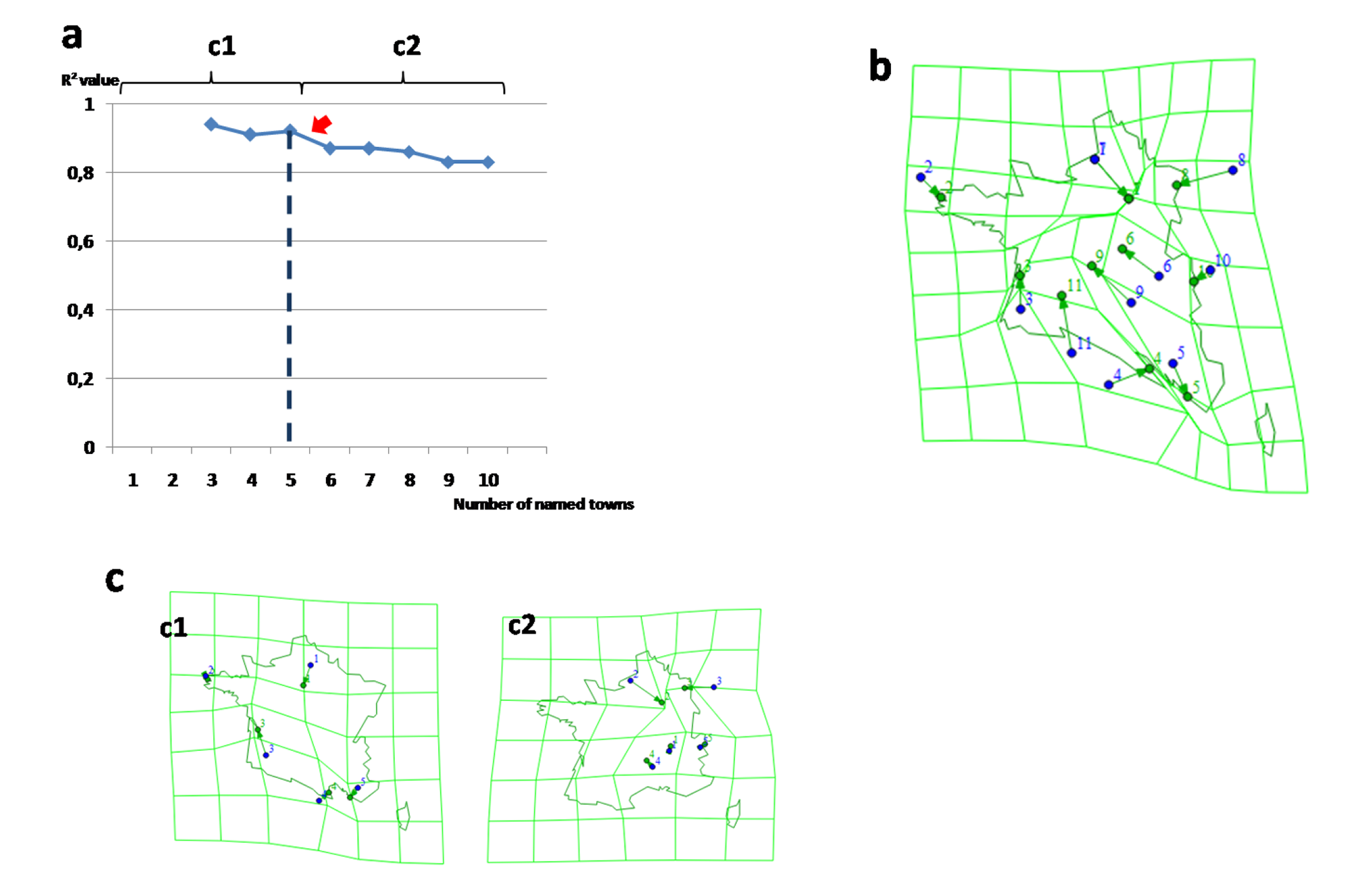

Supplement: Figure S2 — Graphic representation of the cognitive map of France as reflected by gaze positions, in the imagery task, for the subject n°3. (TIF) [file pone.0068560.s002.tif]

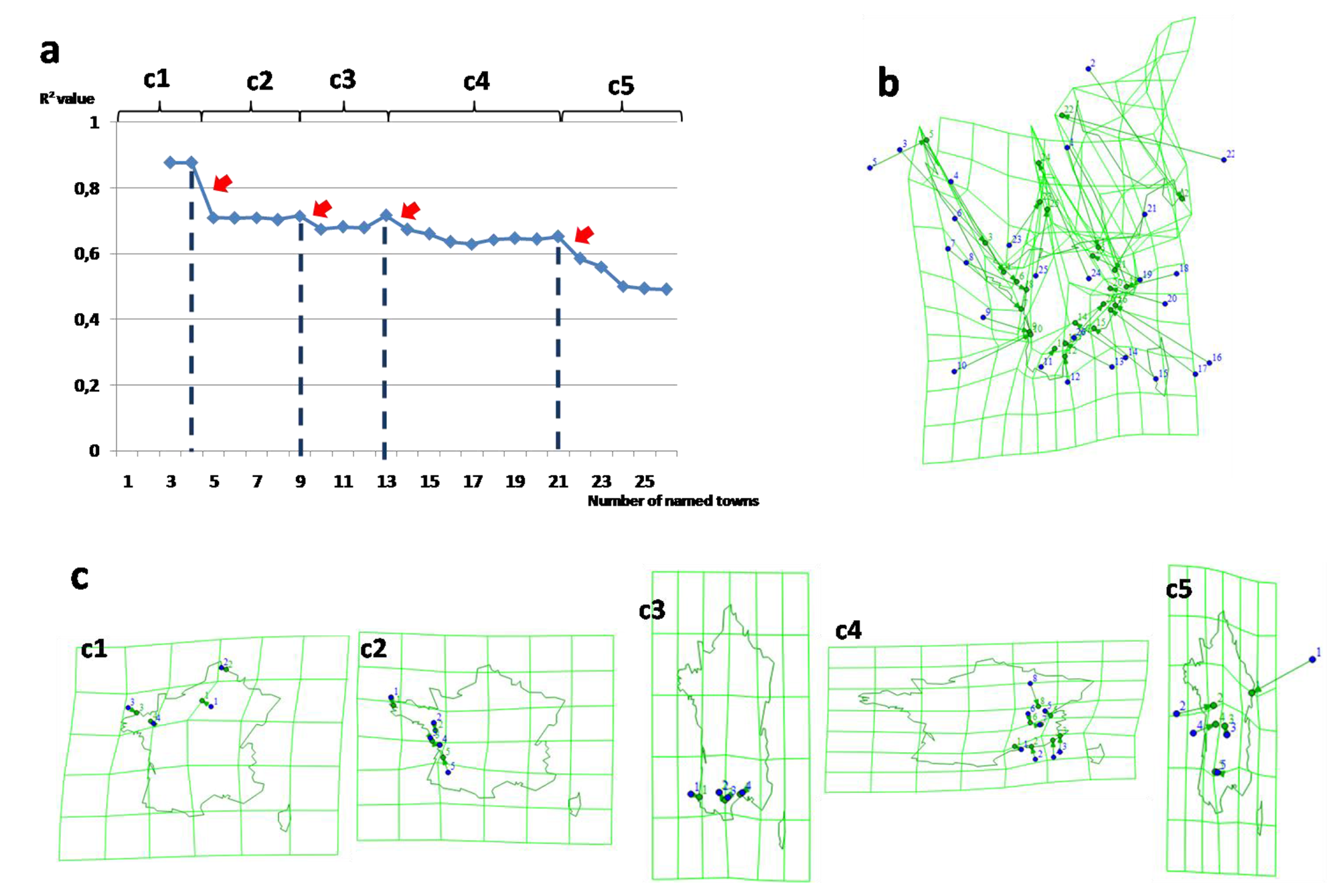

Supplement: Figure S3 — Graphic representation of the cognitive map of France as reflected by gaze positions, in the imagery task, for the subject n°4. (TIF) [file pone.0068560.s003.tif]

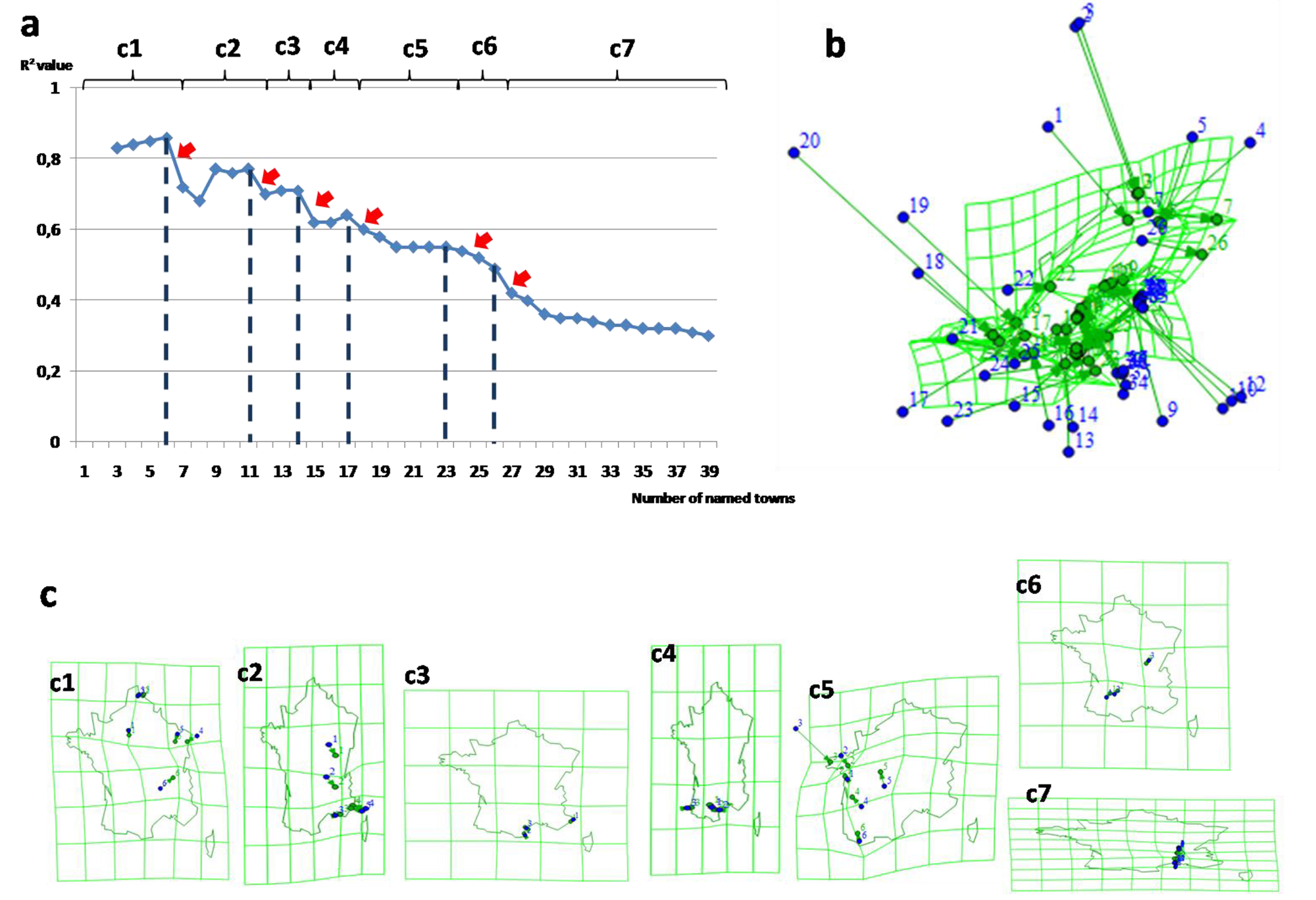

Supplement: Figure S4 — Graphic representation of the cognitive map of France as reflected by gaze positions, in the imagery task, for the subject n°5. (TIF) [file pone.0068560.s004.tif]

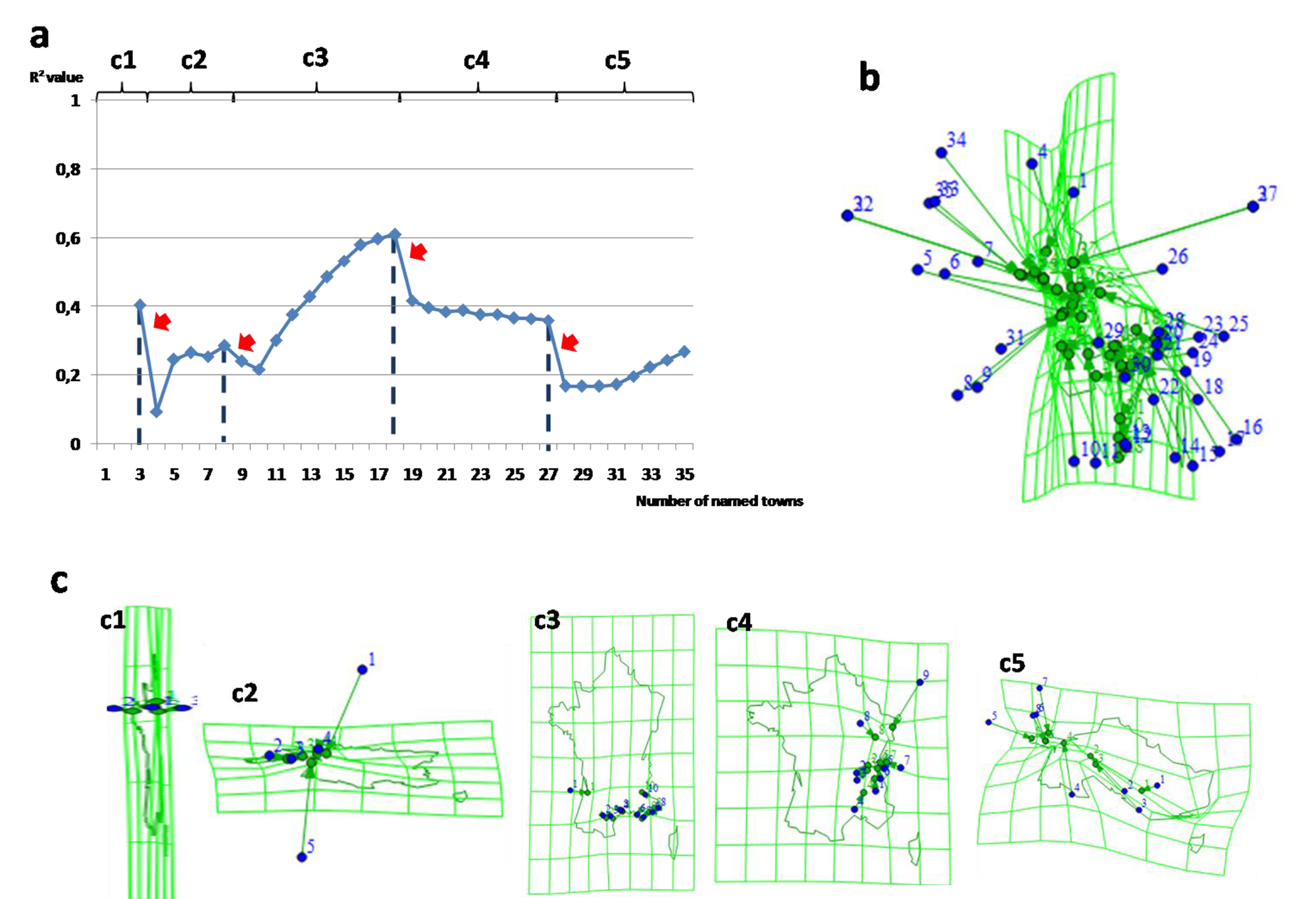

Supplement: Figure S5 — Graphic representation of the cognitive map of France as reflected by gaze positions, in the imagery task, for the subject n°6. (TIF) [file pone.0068560.s005.tif]

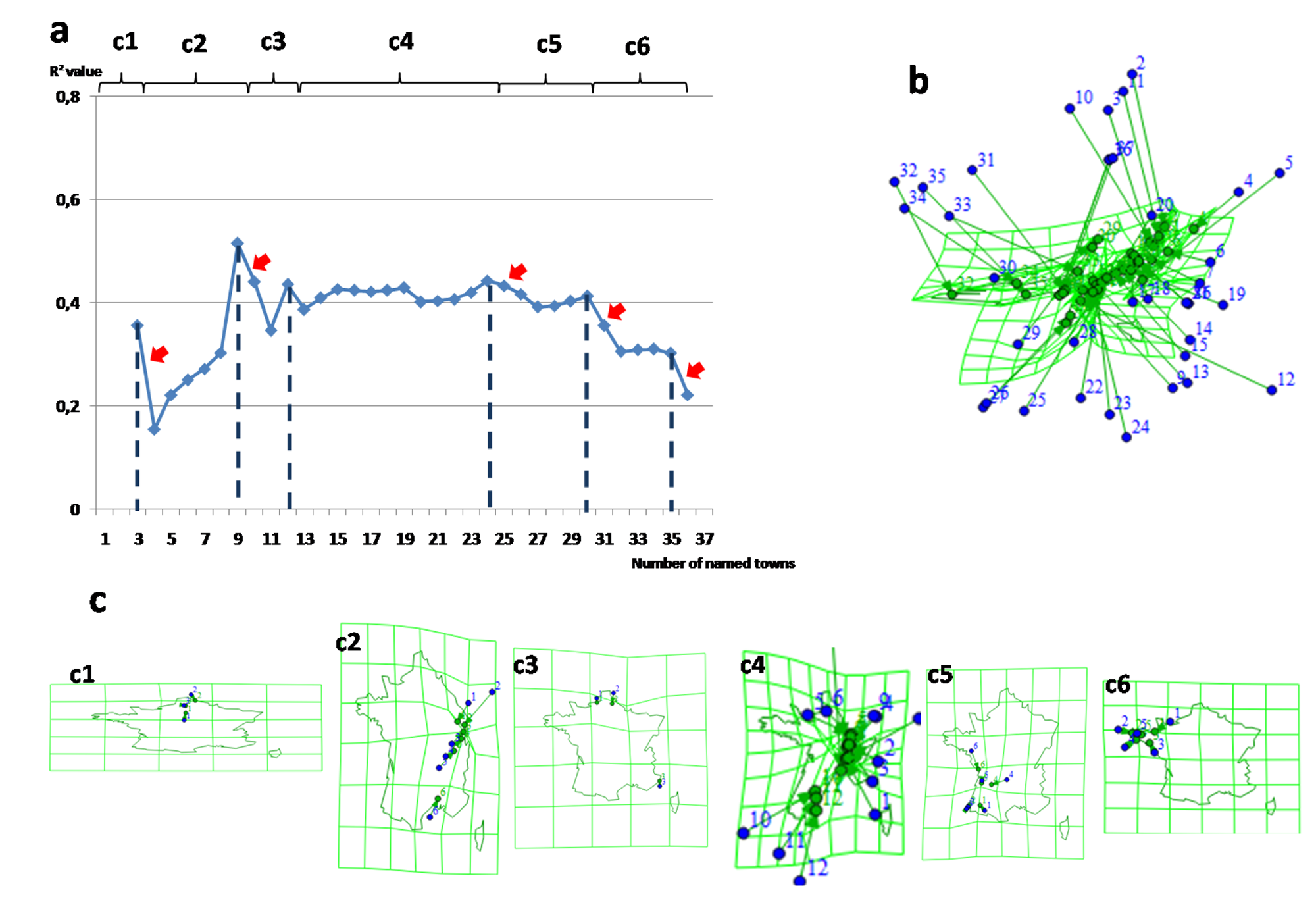

Supplement: Figure S6 — Graphic representation of the cognitive map of France as reflected by gaze positions, in the imagery task, for the subject n°7. (TIF) [file pone.0068560.s006.tif]

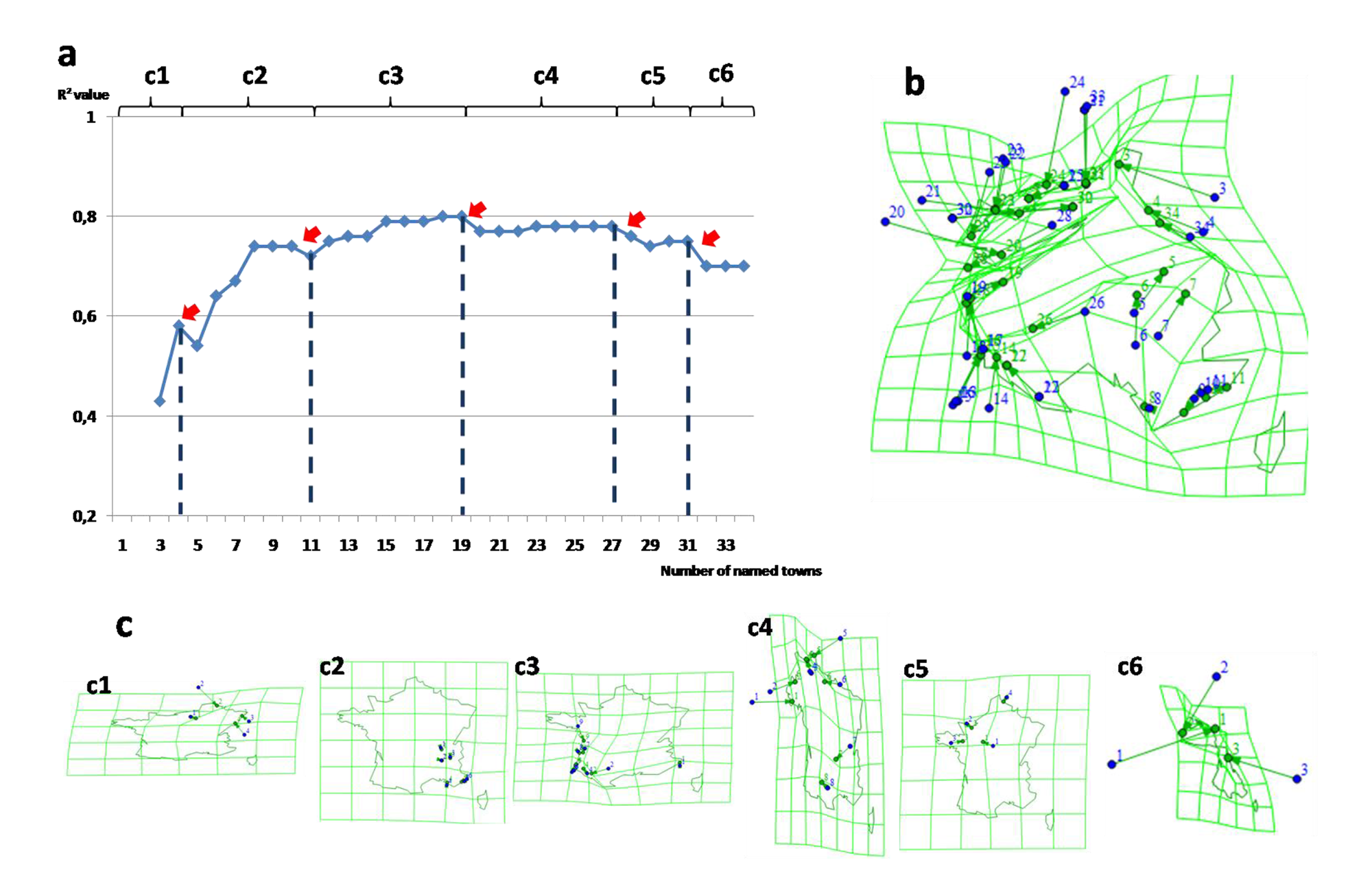

Supplement: Figure S7 — Graphic representation of the cognitive map of France as reflected by gaze positions, in the imagery task, for the subject n°8. (TIF) [file pone.0068560.s007.tif]

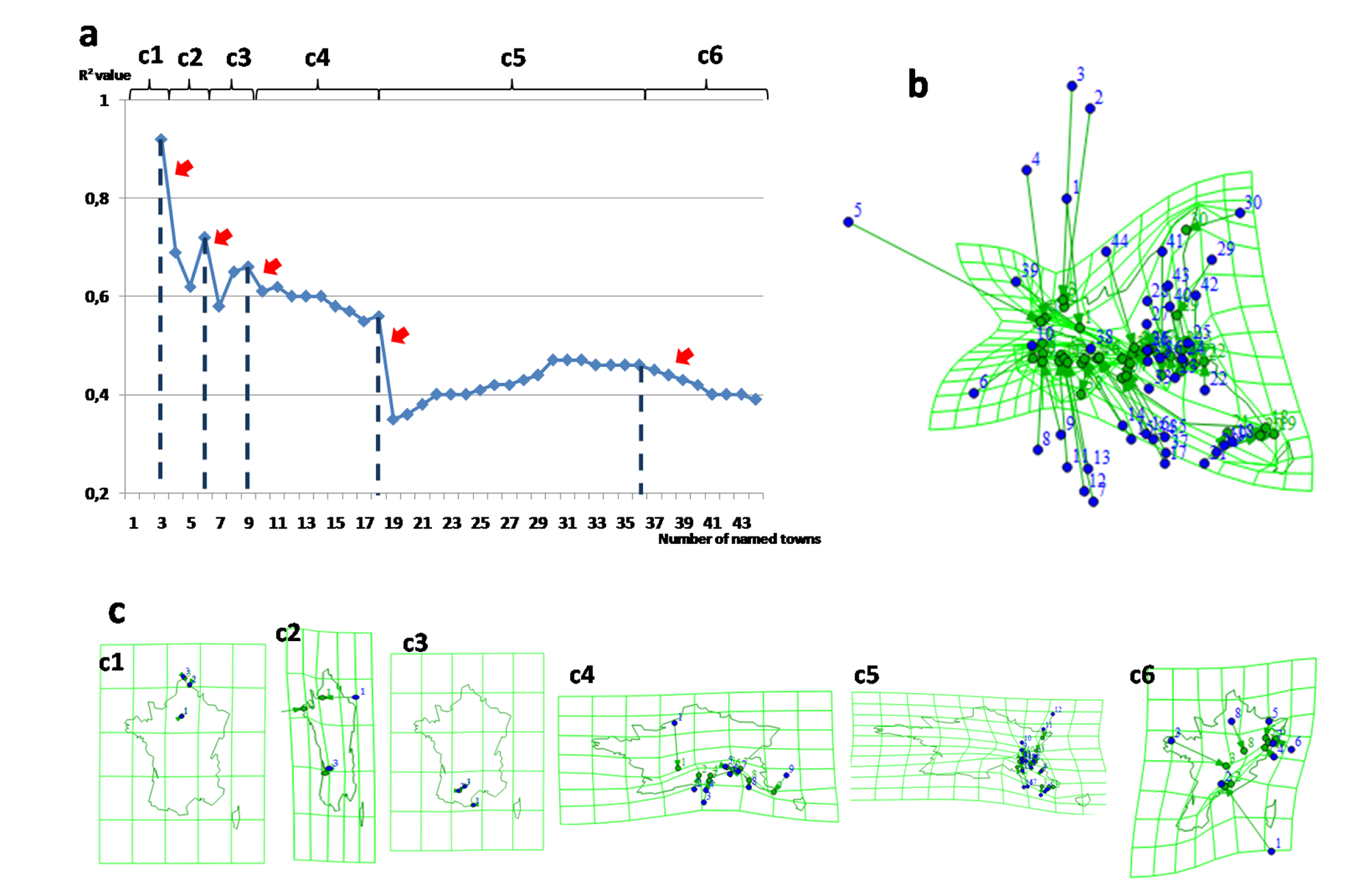

Supplement: Figure S8 — Graphic representation of the cognitive map of France as reflected by gaze positions, in the imagery task, for the subject n°9. (TIF) [file pone.0068560.s008.tif]

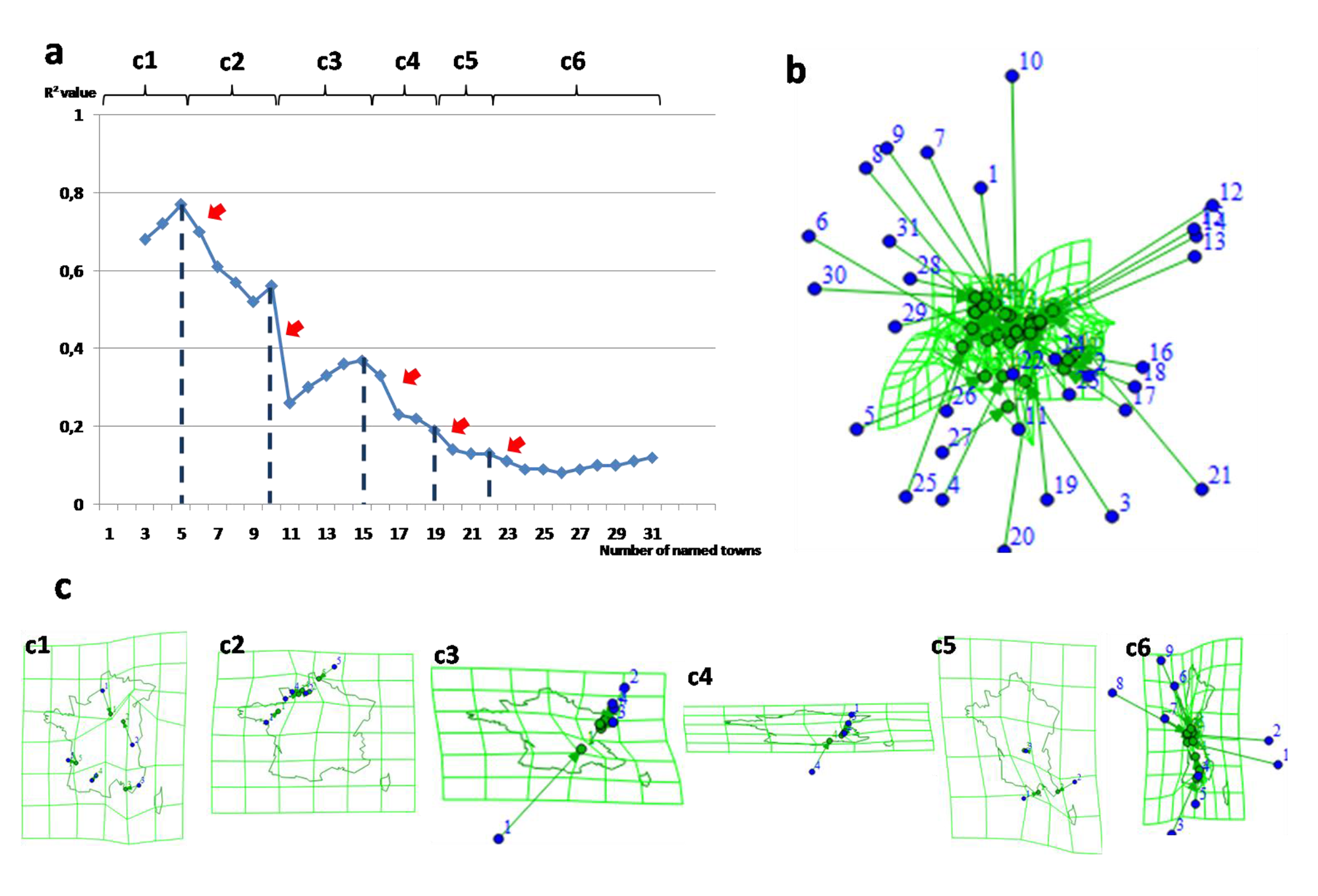

Supplement: Figure S9 — Graphic representation of the cognitive map of France as reflected by gaze positions, in the imagery task, for the subject n°10. (TIF) [file pone.0068560.s009.tif]
